# Effects of an eight-week supervised, structured lifestyle modification programme on anthropometric, metabolic and cardiovascular risk factors in severely obese adults

**DOI:** 10.1186/s12902-015-0038-x

**Published:** 2015-08-01

**Authors:** Catherine Crowe, Irene Gibson, Katie Cunningham, Claire Kerins, Caroline Costello, Jane Windle, Paula M. O′Shea, Mary Hynes, Brian McGuire, Katriona Kilkelly, Helena Griffin, Tim O′Brien, Jenni Jones, Francis M Finucane

**Affiliations:** Bariatric Medicine Service, Galway Diabetes Research Centre, HRB Clinical Research Facility, Galway University Hospital, Galway, Ireland; Croi, the West of Ireland Cardiac Foundation, Heart and Stroke Centre, Moyola Lane, Newcastle, Galway Ireland; Department of Clinical Biochemistry, Galway University Hospitals, Galway, Ireland; School of Psychology, National University of Ireland, Galway, Ireland; Discipline of Health Promotion, National University of Ireland, Galway, Ireland; National Institute of Preventive Cardiology, Galway, Ireland

**Keywords:** Bariatric, Structured lifestyle modification, Cardiovascular risk, CLANN (Changing Lifestyle with Activity and Nutrition) Programme, Nurse-led, Diabetes

## Abstract

**Background:**

Lifestyle modification is fundamental to obesity treatment, but few studies have described the effects of structured lifestyle programmes specifically in bariatric patients. We sought to describe changes in anthropometric and metabolic characteristics in a cohort of bariatric patients after participation in a nurse-led, structured lifestyle programme.

**Methods:**

We conducted a retrospective, observational cohort study of adults with a body mass index (BMI) ≥40 kgm^−2^ (or ≥35 kgm^−2^ with significant co-morbidity) who were attending a regional bariatric service and who completed a single centre, 8-week, nurse-led multidisciplinary lifestyle modification programme. Weight, height, waist circumference, blood pressure, HbA1c, fasting glucose and lipid profiles as well as functional capacity (Incremental Shuttle Walk Test) and questionnaire-based anxiety and depression scores before and after the programme were compared in per-protocol analyses.

**Results:**

Of 183 bariatric patients enrolled, 150 (81.9 %) completed the programme. Mean age of completers was 47.9 ± 11.2 years. 34.7 % were male. There were statistically significant reductions in weight (129.6 ± 25.9 v 126.9 ± 26.1 kg, *p* < 0.001), BMI (46.3 ± 8.3 v 44.9 ± 9.0 kgm^−2^, *p* < 0.001), waist circumference (133.0 ± 17.1 v 129.3 ± 17.5 cm in women and 143.8 ± 19.0 v 135.1 ± 17.9 cm in men, both *p* < 0.001) as well as anxiety and depression scores, total- and LDL-cholesterol and triglyceride levels, with an increase in functional capacity (5.9 ± 1.7 v 6.8 ± 2.1 metabolic equivalents of thermogenesis (METS), *p* < 0.001) in completers at the end of the programme compared to the start. Blood pressure improved, with reductions in systolic and diastolic blood pressure from 135 ± 16.2 to 131.6 ± 17.1 (*p* = 0.009) and 84.7 ± 10.2 to 81.4 ± 10.9 mmHg (*p* < 0.001), respectively. The proportion of patients achieving target blood pressure increased from 50.3 to 59.3 % (*p* = 0.04). The proportion of patients with diabetes achieving HbA1c <53 mmol/mol increased from 28.6 to 42.9 %, *p* = 0.02.

**Conclusions:**

Bariatric patients completing an 8 week, nurse-led structured lifestyle programme had improved adiposity, fitness, lipid profiles, psychosocial health, blood pressure and glycaemia. Further assessment of this programme in a pragmatic randomised controlled trial seems warranted.

## Background

Consistent with global trends, the prevalence of overweight and obesity has increased steadily in Ireland over the past three decades [1, 2]. This is a public health concern because of the associated morbidity and mortality from diseases like type 2 diabetes, for which the population attributable risk from obesity is 50 to 80 % [3, 4]. There is also a significant health economic burden associated with obesity, which accounts for €1.1 billion of public spending per annum in Ireland [5]. Public health strategies to reduce calorie intake and increase physical activity have not been successful to date. The population shift in body mass index (BMI) has led to a particularly dramatic rise in the prevalence of severe obesity [6]. For these individuals (conventionally defined as those with a BMI ≥40 kg m^−2^ or ≥35 kg m^−2^ with co-morbidities such as type 2 diabetes), relatively intensive “bariatric” interventions need to be adopted [7]. These include intensive dietary restriction [8] and bariatric surgery [9]. In a US cohort of bariatric surgical patients, the prevalence of hypertension was 49 %, type 2 diabetes was 28 % and dyslipidaemia was 46 % at baseline, which was reduced or resolved by 68, 75 and 71 % respectively after a mean post-surgical follow up of 34 months, with a 40 % relative reduction in cardiovascular risk in that time [10]. While bariatric surgical interventions are efficacious and cost effective [11], they are also invasive, expensive and unsuitable for a significant proportion of bariatric patients.

Lifestyle modification is the cornerstone of the therapeutic approach to the obese patient. Several studies have confirmed the benefits of structured lifestyle interventions in different patient subgroups, including those with non-diabetic hyperglycaemia [12–14], prevalent cardiovascular disease [15] or established type 2 diabetes [16]. However, studies of such interventions in bariatric patients are limited and often include meal replacement regimes, which can be very effective in the short term but are not designed to induce sustained dietary changes and are associated with low retention rates [17]. In one US study of 130 bariatric patients undergoing a twelve-month diet and physical activity programme, weight loss at 1 year was 11 kg, with significant reductions in waist circumference, visceral and hepatic fat, blood pressure and insulin resistance, with a study retention rate of 78 % [18]. However, there was still a meal replacement component to this trial and to our knowledge, no previous study has examined the effects of a multidisciplinary lifestyle modification programme that does not include a prescribed diet or meal replacement regimen in bariatric patients.

As part of the recent establishment of a regional bariatric service in the West of Ireland, we have adopted an 8 week structured lifestyle modification programme known as the CLANN (Changing Lifestyle with Activity and Nutrition) for patients with severe obesity. This programme incorporates some key principles which have already been associated with successful reduction in anthropometric and metabolic cardiovascular risk factors in adults with prevalent cardiovascular disease [19, 20]. Our objective in this study was to measure the effect of this programme on relevant cardiovascular outcomes. In achieving that objective, we hope to facilitate the more robust design of a randomised controlled trial to determine the programme’s effectiveness in bariatric patients.

## Methods

The study population included bariatric patients ≥18 years old with a BMI ≥40 kg m^−2^ (or ≥35 with co-morbidity) who were willing to attend a community-based facility where the CLANN programme is delivered for 2 h per week for 8 weeks. Each patient was referred after careful assessment for suitability by the hospital-based, multidisciplinary bariatric team. Patients with poorly controlled diabetes or hypertension, symptoms suggestive of untreated ischaemic heart disease, an inability to walk 10 m or those deemed unlikely to attend for the full programme (e.g. frequent clinic non-attendance) were excluded from the programme.

The 8-week programme focused on weight management as part of a comprehensive cardiovascular risk reduction strategy. The main emphasis of the programme is on lifestyle modification, namely healthy food choices, weight reduction, physical activity and smoking cessation. We based our definition of “healthy food choices” on the European guidelines on healthy eating for cardiovascular disease prevention [21]. Specifically, these stipulate saturated fatty acids <10 % of total daily energy intake, trans-unsaturated fatty acids <1 % of total daily energy intake, salt consumption <5 g/ day, fibre intake 30–45 g/ day, fruit >200 g/ day (2–3 servings), vegetables >200 g/ day (2–3 servings), fish at least twice per week, (one “oily”) and alcohol consumption <20 g/ day for men and <10 g/ day for women. The Irish “Food Pyramid” (published by the Irish Government’s Department of Health in 2012) was used as a visual tool to encourage the adoption of these dietary guidelines and to describe portion sizes to the participants. We sought to encourage a cardioprotective diet and to achieve an energy deficit of 600 kcal/ day, in order to achieve a target weekly weight loss of 0.5 kg.

Initially, all patients received an individualised assessment by the multidisciplinary team (nurse, dietitian, physiotherapist, physical activity specialist). This included recording use of cardioprotective medications such as antihypertensive and antiplatelet agents and statins. The assessment also sought to identify individual priorities and needs and to explore beliefs, barriers and motivators to change. Following this assessment, patients attended the 8-week programme, consisting of a group-based exercise programme combined with educational workshops, lasting 2 h each week. Effective exercise and physical activity strategies which were specific, measurable, achievable, realistic and timed were also emphasised. These were initiated in the programme without specialist gym equipment with the objective of the patient maintaining these behaviours in the longer term after completion of the programme. While the exercise sessions were group-based for maximum efficiency, each patient had an individualised programme based on an assessment of their needs by the physiotherapist and exercise physiologist. The curriculum of eight theory-based health promotion workshops incorporated a module on “understanding cardiovascular disease”, two modules on “eating for a healthy shape” (emphasising dietary modification techniques such as carbohydrate counting, food label interpretation and portion size calculation) and a module each on “exercising for life”, “reading and understanding food labels”, “stress management”, “dealing with the psychological issues of obesity” (including mindfulness) and “maintaining a healthy lifestyle”.

Individualised goal setting occurred at the initial assessment and then continued throughout the programme. Motivational interviewing was used to support patients in progressing through the stages of behaviour change, by enhancing intrinsic motivation, autonomy and self-efficacy. All patients were given a personal record card which they were encouraged to use on a weekly basis to record their goals and track their progress in relation to weight, BMI, blood pressure, lipid profile and for patients with diabetes, HbA1c and glucose levels.

At initial assessment, dietary habits, physical activity levels (7-day physical activity recall), functional capacity (Incremental Shuttle Walk Test [22], (ISWT) from which a Heart Rate Walking Speed Index (HRWSI) was derived) and psychosocial measures (questionnaire-based anxiety and depression scores) were recorded. Specifically, the Hospital Anxiety and Depression Scale (HADS) [23], EQVAS (European Quality of Life Questionnaire, Visual Analogue Scale) [24] and Dartmouth COOP [25] questionnaires were administered. Weight was measured using a Seca® 877 scale and height with a Seca® Leicester stadiometer. Blood pressure was measured with an Omron® 705IT oscillometric device in both arms after the patient was sitting comfortably for five minutes. All of these measures were repeated following completion of the programme. The outcome targets were based on the 2012 European Society of Cardiology (ESC) prevention guidelines [21]. Specifically, the blood pressure target was 140/ 90 mmHg (140/ 85 in patients with type 2 diabetes) while the lipid targets were total cholesterol <5 mmol/l, LDL cholesterol <3 mmol/l and triglycerides <1.7 mmol/l. The study was approved by the Galway Clinical Research Ethics Committee (the ethics committee for Galway University Hospitals). Patients signed written informed consent for their data to be used in the study.

### Laboratory analysis

All blood samples were analysed locally in the Galway University Hospitals’ Department of Clinical Biochemistry (certified to ISO 15189 2007 accreditation standard). HbA1c was measured with HPLC (Menarini® HA8160 auto-analyzer). Total cholesterol was measured using the CHOP-PAP method. High density lipoprotein (HDL)-cholesterol and triglycerides were measured using the enzymatic and the GPO-PAP methods, respectively (Roche COBAS® 8000 modular analyzer). Low density lipoprotein (LDL)-Cholesterol was derived with the Friedewald equation [26].

### Statistical analysis

Differences between outcomes at the start and the end of the programme for patients who completed the programme were assessed using the paired exact test for categorical variables, the paired *t*-test for normally distributed continuous variables and the Wilcoxon matched pairs test for non-normally distributed continuous variables. All analyses were performed using SPSS version 21.

## Results

Of 183 patients enrolled, 150 (81.9 %) completed the programme. Their baseline socio-demographic characteristics are shown in Table 1. Mean age was 47.9 ± 11.3 years. 57 (31 %) had type 2 diabetes at baseline. Overall, of 136 (92.7 %) of participants who provided information about their employment status, 78 (57.4 %) were unemployed. Most participants were “White Irish”, though 51 (34 %) did not provide information about their ethnicity. Similarly, 47 participants (31.3 %) did not provide information about their educational background. The baseline and follow-up anthropometric and metabolic characteristics of programme completers are presented in Table 2. Patients who completed the programme had statistically significant reductions in weight, BMI, waist circumference and blood pressure. Changes in blood pressure were greater in those who had suboptimal blood pressure at baseline, defined as >140/85 and >140/90 mmHg in patients with and without type 2 diabetes, respectively [27]. The proportion of patients with blood pressure readings at target increased from 50.3 % at baseline to 59.3 % at follow-up, *p* = 0.04. There were reductions also in self-reported depression and anxiety levels and some, but not all components of the lipid profile. HbA1c decreased slightly in the whole group, with greater reductions in those with type 2 diabetes. The proportion of patients with type 2 diabetes achieving optimal control (HbA1c <53 mmol/mol or 7 %) increased from 28.6 % at baseline to 42.9 % at follow-up, *p* = 0.02. The proportion of patients achieving targets for moderate intensity aerobic activity of 30 min five times per week [28] increased from 4.0 % at baseline to 38.4 % at follow-up, *p* < 0.001. There were significant increases in various indices of aerobic fitness and functional status, as shown in Table 1. Lastly, changes in medication usage were minimised to the greatest extent possible (Table 2). One patient stopped their calcium channel blocker and one patient started an ACE inhibitor, otherwise no changes were made to these medications.

**Table 1:** Baseline socio-demographic characteristics of 150 severely obese adults completing the Croi CLANN programme

	*N*	Proportion (%)
Demographic variables		
Female	98/ 150	65.3
Current smoker	18/ 150	12
Living with partner	91/ 144	60.7
Prevalent diabetes	53/ 148	35.8
Employment status (*N* = 136)		
Employed full time	38	27.9
Employed part time	13	9.6
Self-employed full time	6	4.4
Self-employed part time	1	0.7
Retired	17	12.5
Unemployed	25	18.4
Looking after family	20	14.7
Student	9	6.6
Permanently sick	2	1.5
Temporarily sick	2	1.5
Other	3	2.2
Ethnicity (*N* = 99)		
White / White Irish	98	99
Other ethnicity	1	1
Education level (*N* = 103)		
Primary	23	22.3
Secondary	38	36.9
College / University	42	40.8

Proportions are expressed as a percentage of the total number of participants for which data were available for that variable

**Table 2:** Baseline and follow-up anthropometric and metabolic characteristics of 150 severely obese adults completing the Croi CLANN programme

Variable	*N*	Baseline	Follow-up	Difference		*P*-value
		Mean ± SD	Mean ± SD	Mean	95 % Confidence Interval	
Weight (kg)	150	129.6 ± 29.5	126.9 ± 26.1	−2.7	[−3.4, −2.0]	<0.001
BMI (kg/m^2^)	150	46.3 ± 8.3	44.9 ± 9.0	−1.4	[−2.1, −0.7]	<0.001
Waist (cm) - women	93	133.3 ± 17.1	129.3 ± 17.5	−3.7	[−4.9, −2.4]	<0.001
Waist (cm) – men	50	143.8 ± 19.0	135.1 ± 17.9	−8.0	[−10.5, −5.4]	<0.001
SBP (mmHg)	144	135.0 ± 16.2	131.6 ± 17.1	−3.3	[−5.8, −0.8]	0.009
DBP (mmHg)	144	84.7 ± 10.2	81.4 ± 10.9	−3.2	[−4.9, −1.6]	<0.001
SBP (mmHg) ^a^	71	145.5 ± 14.8	138.2 ± 17.3	−7.3	[−11.3, −3.3]	<0.001
DBP (mmHg)^a^	71	91.7 ± 8.0	85.7 ± 11.3	−6.0	[−8.5, −3.5]	<0.001
HbA1c (mmol/mol)	114	42 (37, 60)	41 (37, 53)	0	[−2, 0]	0.007
HbA1c (mmol/mol)^b^	51	61 (49, 71)	59 (45, 66)	−3	[−5, 0]	0.006
Total Cholesterol (mmol/L)	145	4.7 ± 1.1	4.5 ± 1.0	−0.2	[−0.3, −0.1]	0.001
LDL Cholesterol (mmol/L)	138	2.7 ± 1.0	2.6 ± 0.9	−0.1	[−0.2, 0.0]	0.05
HDL Cholesterol (mmol/L)	145	1.2 ± 0.3	1.1 ± 0.3	−0.02	[−0.04, 0.01]	0.16
Triglycerides (mmol/L)	145	1.6 (1.1, 2.2)	1.4 (1.0, 1.9)	−0.1	[−0.2, −0.01]	<0.001
Total Cholesterol (mmol/L) ^c^	62	5.8 ± 0.8	5.2 ± 0.9	−0.6	[−0.8, −0.4]	<0.001
LDL Cholesterol (mmol/L) ^c^	62	3.8 ± 0.8	3.4 ± 0.7	−0.4	[−0.6, −0.2]	<0.001
Triglycerides (mmol/L)^c^	62	2.2 (1.9, 2.8)	1.9 (1.4, 2.8)	−0.4	[−0.6, −0.03]	<0.001
Metres scored on ISWT (m)	133	295 ± 130	350 ± 153	53	[41, 66]	<0.001
METs achieved on ISWT (MET)	133	3.5 ± 0.9	3.8 ± 1.3	0.4	[0.3, 0.6]	<0.001
Estimated MET max (MET)	133	5.9 ± 1.7	6.8 ± 2.1	0.9	[0.6, 1.1]	<0.001
HRWSI	133	16.9 ± 4.3	15.5 ± 3.6	−1.4	[−1.9, −0.9]	<0.001
Functional limitations score	83	20.5 ± 5.5	22.5 ± 5.3	2.0	[1.1, 2.9]	<0.001
Anxiety score	110	7 (5, 11)	7 (4, 10)	−1	[−2, 0]	<0.001
Depression score	110	8 (5, 11)	4 (2, 7)	−2	[−3, −1]	<0.001
Dartmouth Coop score	110	26 ± 6	23 ± 7	−3	[−4, −2]	<0.001
EQ-VAS	110	53 ± 22	60 ± 20	8	[4, 11]	<0.001
Antiplatelet agents (%)	150	27.4	27.4	0	[−0.7, 0.7]	1.0
Statins (%)	150	34.3	34.3	0	[−0.7, 0.7]	1.0
ACEI/ ARB (%)	150	38.5	39.2	0.7	[−2.4, 3.8]	1.0
Betablockers (%)	150	17.6	17.6	0	[−0.7, 0.7]	1.0
Calcium channel blockers (%)	150	22.6	21.9	−0.7	[−2.7, 1.3]	1.0

Data are presented as mean ± standard deviation or as median (interquartile range)

^a^Denotes subgroup with elevated blood pressure at baseline assessment (>140/85 mmHg with diabetes or >140/90 with no diabetes)

^b^Denotes subgroup with prevalent type 2 diabetes at baseline

^c^Denotes subgroup not meeting that specific lipid subtype target at baseline [21]

In order to determine whether the response to the intervention was different for men and women, we used regression modelling with the follow-up measure of the variable of interest being the outcome and sex as the exposure, adjusting for the baseline measure of that variable. Overall, there were differential responses for only a small number of outcomes. Specifically, men had a greater reduction in waist circumference than women (−3.4 [−6.0, −0.8] cm, *p* = 0.009). Men had statistically significantly greater improvements in some but not all indices of aerobic fitness. Their reduction in heart rate walking speed index (HRWSI) was greater (−1.0 [−1.8, −0.2], *p* = 0.02) and their increase in estimated MET max was greater (0.6 [0.1, 1.0] METs, *p* = 0.02), though changes in metres scored on ISWT and METs achieved were not statistically significantly different by sex. After adjusting for baseline values, triglycerides were 12 % lower in men than in women at follow up (*p* = 0.02). Conversely the reduction in systolic blood pressure was lower for men than for women, such that the decrease in women was 6.6 [1.8, 11.4] mmHg (*p* = 0.007) more than for men.

We also sought to determine whether there were any differences at baseline in patients who completed the programme compared to those who dropped out and did not attend for follow-up assessment. Compared to non-completers, completers were older (47.9 ± 11.2 versus 40.7 ± 12.9 years, *p* = 0.003), were more likely to be men (34.7 versus 10.3 %, *p* = 0.008) and were more likely to have diabetes (35.8 versus 13.8 %, *p* = 0.03). There were no differences in smoking, employment, ethnicity or educational status, nor were there differences in anthropometric measures such as weight, BMI, waist circumference or blood pressure. As anticipated given the above differences, HbA1c was higher in completers than non-completers at baseline (47.9 ± 15.5 versus 39.7 ± 5.1 mmol/mol, *p* = 0.02) and HDL cholesterol was lower (1.15 ± 0.28 versus 1.29 ± 0.38, *p* = 0.03), though there was no difference in other components of the lipid profile. Also, medication usage was higher at baseline in completers, for statins (33.6 versus 11.1 %, *p* = 0.02), antiplatelets (27.9 versus 3.6 %, *p* = 0.004), ACEI/ ARBs (38.9 versus 15.4 %, *p* = 0.03) and calcium channel blockers (22.5 versus 0 %, *p* = 0.03) but not for betablockers (7.4 versus 17.5 %, *p* = 0.26). Completers had less self-reported anxiety at baseline (7 [5, 11] versus 9 [6, 12], *p* = 0.04), though other psychometric measures were not different. They achieved a shorter distance on the incremental shuttle walk test (295 ± 130 versus 364 ± 106, *p* = 0.01), though there were no differences in other measures of fitness. We sought also to determine whether the response to the programme was different according to diabetes status. Compared to non-diabetic patients, patients with diabetes who completed the programme had a greater reduction in waist circumference (−3.2 [−5.6, −0.7] cm, *p* = 0.01), though changes in weight and BMI were not different. There was a trend to a greater reduction in systolic blood pressure in those without diabetes of 4.8 mmHg [0.0, 9.6], *p* = 0.05, but not in diastolic blood pressure, lipid profile or any measures of fitness. Changes in psychometric scores were no different by diabetes status (data not shown).

## Discussion

These findings confirm that a structured lifestyle modification programme for carefully selected bariatric patients is feasible and safe and leads to statistically significant improvements in several cardiovascular risk-related outcomes. The retention rate of 81.9 % compares favourably with similar lifestyle intervention studies in this patient group [17, 18]. While the overall effect size of the programme in completers was relatively modest, the reduction in BMI of 1.4 kgm^−2^ is identical to that seen in cardiovascular patients attending the Croi “MyAction” programme at the same facility [19]. We anticipated that blood pressure and lipid profiles might not change to the same extent in “Clann” as in “MyAction”, because we sought to minimise any changes in medications during this programme to the greatest extent possible, whereas in the Croi “MyAction” programme the proportions of patients on antiplatelets, renin-angiotensin related antihypertensives and calcium channel blockers increased by 4.6, 4.9 and 5.6 %, respectively while the proportion on statins increased from 40.3 to 65.1 %. The improvements we have observed in the “Clann” programme have not arisen because of increased prescribing of medications which lower cardiovascular risk and are likely therefore to have been driven by the programme itself.

The relatively small changes in body weight and BMI, particularly in the context of the very abnormal level of adiposity these patients have, might be seen as an indicator that the overall impact on health of this programme is not strong enough. While pervading dogma suggests that any weight loss is good, recent guidelines state that bariatric patients need to lose 15 % or more of their body weight to have a meaningful improvement in their health [29]. However, favourable changes in body composition such as reduced fat mass with concurrent increases in lean mass are unlikely to be identified through changes in weight or BMI, so important and meaningful improvements may still have occurred [30]. Moreover, fitness is a far more relevant index of overall wellness and cardiovascular risk than fatness [31, 32]. The mean increase of 15 % in aerobic capacity (estimated MET max) of completers was a strong indicator that the programme is in fact effective and if sustained, might lead to a substantial reduction in mortality [33, 34]. The improvements in depression scores and components of the lipid profile are also clearly relevant and important.

This study has a number of limitations. Firstly, it is a retrospective, post-hoc cohort analysis without a pre-specified primary outcome or a control group, so while the findings provide a useful indication for effect size of this programme on various outcomes, we cannot determine its effectiveness. Out of necessity we have only measured changes in outcomes in patients who attended for follow-up assessments. These are likely to represent a more motivated subgroup than non-completers, which would lead to an overestimation of the potential benefits of the intervention compared to an analysis which included data from patients who did not complete the intervention. Completers were older, more likely to be male and have diabetes, were taking more medications and may have been less fit, but we don’t believe these differences with non-completers account exclusively for the changes we have observed after participation in the programme. It is difficult to refine a target population for recruitment to the programme on the basis of results reported herein, but larger scale studies might help to identify patients who are likely to remain in the intervention and benefit from it. Notwithstanding the differences in the response to the intervention for some outcomes by sex and diabetes status, these weren’t strong or consistent enough to suggest that the intervention should be limited to specified subgroups of patients.

There may have been a Hawthorne effect, whereby outcomes were different by virtue of the fact that they were being measured twice [35]. For example patients may have felt it polite to report that they were feeling less depressed or may have made more of an effort on their ISWT at the second attempt. However, objective improvements in the HRWSI, several metabolic variables and validated questionnaires are unlikely to be due entirely to measurement effect, we believe. As these patients were carefully selected for inclusion by a hospital-based regional bariatric multidisciplinary team, the results may not be generalisable to the broader population of adults with severe obesity. For some variables, there was a relatively high proportion of missing data. For example, the functional limitations score was measured before and after the programme in only 55 % of completers, while 73 % completed questionnaires and 76 % had HbA1c measured at both visits. This may have introduced bias to the results. A prospectively designed pragmatic randomised controlled trial, with blinded assessment, would address many of these limitations. The study has a number of strengths in addition to the high patient retention rate. The programme was delivered in a consistent way to all patients by experienced staff. Adherence to a predefined curriculum of teaching objectives during the health-promotion workshops is likely to have enhanced the integrity of the programme. Similarly, the assessments were comprehensive and were completed according to standardised protocols. Medication usage for hypertension, dyslipidaemia and diabetes was recorded carefully at the start and end of the programme.

## Conclusions

In a well-defined cohort of predominantly white adult bariatric patients attending a regional obesity referral centre who were identified as highly motivated and suitable for a nurse-led structured lifestyle modification programme, the programme led to significant improvements in anthropometric, metabolic and cardiovascular risk variables over 10 weeks. Given that the programme appears feasible, safe and acceptable to patients, with a high retention rate and equivalent improvements to similar interventions in other cohorts of high risk individuals, future studies assessing the impact of structured lifestyle modification in bariatric patients could use it as a framework. The paucity of effective therapeutic strategies apart from bariatric surgery and meal replacement diets for these patients justify the formal assessment of this programme in a randomised controlled trial. Future studies might include more detailed patient phenotyping to determine changes not just in weight and BMI but in fat- and lean- mass and other indices of body composition. Additionally, future studies might examine proximal metabolic outcomes such as insulin sensitivity which are linked to cardiovascular risk. These studies will need to focus on more complete acquisition of socio-demographic and functional limitation questionnaire data.

## Abbreviations

ACEI: Angiotensin converting enzyme inhibitor
ARB: Angiotensin receptor blocker
BMI: Body mass index
CLANN: Changing lifestyle with activity and nutrition
DBP: Diastolic blood pressure
EQ-VAS: European quality of life questionnaire, visual analogue scale
HDL: High density lipoprotein cholesterol
HRWSI: Heart rate walking speed index
ISWT: Incremental shuttle walk test
LDL: Low density lipoprotein cholesterol
METS: Metabolic Equivalents (of Thermogenesis)
SBP: Systolic blood pressure
